# Synthesis of Proposed Structure of Aaptoline A, a Marine Sponge-Derived 7,8-Dihydroxyquinoline, and Its Neuroprotective Properties in *C. elegans*

**DOI:** 10.3390/molecules26195964

**Published:** 2021-10-01

**Authors:** Soobin Kim, Wooin Yang, Dong Seok Cha, Young Taek Han

**Affiliations:** 1 College of Pharmacy, Dankook University, Cheonan 31116, Chungnam, Korea; rue42351@naver.com; 2 College of Pharmacy, Woosuk University, Wanju-gun 55338, Jeonbuk, Korea; benefinn@naver.com

**Keywords:** aaptoline A, 7,8-dihydroxyquinoline, silver-catalyzed cycloisomerization, dopaminergic neuroprotection, Parkinson’s disease

## Abstract

A concise and efficient synthesis of the proposed structure of aaptoline A, a 7,8-dihydroxyquinoline derived from a marine sponge, was accomplished in seven steps with a 52% overall yield. A key feature of the synthesis is the high-yielding Ag(I)-catalyzed cycloisomerization of the *N*-propargylaniline precursor to afford the quinoline carboxylate skeleton from acid-labile methyl aminobenzoate. However, the spectral data of the synthesized aaptoline A were not consistent with those of previous studies. The structure of the synthesized aaptoline A was confirmed by combined 2D NMR analysis. Additional studies on the bioactivity of the synthesized aaptoline A revealed that it has the ability to protect dopaminergic neurons against MPP^+^-induced neurotoxicity in *C. elegans*. In addition, impaired food-sensing ability and travel distance capability in *C. elegans* were significantly ameliorated by aaptoline A treatment, suggesting that aaptoline A can protect dopaminergic neurons both morphologically and functionally.

## 1. Introduction

8-Hydroxyquinoline is considered one of the most important heterocyclic privileged scaffolds in the field of drug discovery and development, as 8-hydroxyquinoline derivatives exhibit a range of biological activities, including antioxidant effects and, significantly, the formation of coordination complexes with transition metal cations. For instance, 8-hydroxyquinoline is an excellent scaffold with therapeutic effects against cancers, neurodegenerative diseases, infectious diseases owing to its strong chelating property with various cations, including Fe^2+^, Fe^3+^, and Zn^2+^ [1]. For this reason, 8-hydroxyquinolines and their analogs, such as 7,8-hydroxyquinolines, have attracted the attention of synthetic and medicinal chemists [1,2,3,4].

Parkinson’s disease (PD) is the second most common neurodegenerative disease and is caused by the progressive loss of dopaminergic (DA) neurons in the substantia nigra pars compacta. Although the etiology of PD remains unclear, oxidative stress is believed to play an important role in DA neurodegeneration [5]. Indeed, many animal model studies of PD have been based on exposure to mitochondrial neurotoxins such as 1-methyl-4-phenylpyridinium (MPP^+^), which induces the selective death of DA neurons by increasing oxidative stress [6]. To date, several PD models have been developed, ranging from mammalian to invertebrate systems, and the nematode *Caenorhabditis elegans* has drawn attention as an excellent experimental model for the study of PD because of its highly conserved DA system and advantages in high-throughput drug screening approaches [6,7].

**Figure 1:**
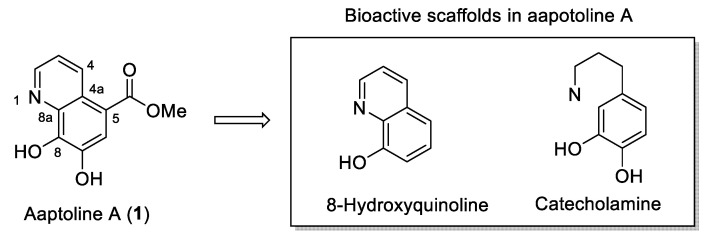
Proposed structure of aaptoline A (**1**) and its embedded privileged scaffolds.

Aaptoline A (**1**) was originally reported as a natural 7,8-dihydroxyquinoline alkaloid isolated from the marine sponge *Aaptos suberitoides* by Kudo et al. in 2014 [8]. Notably, a Chinese research group also recently appointed a different 7,8-hydroxyquinoline alkaloid derived from *Aaptos aaptos* as aaptoline A [9]. **1** was expected to have therapeutic properties against neurodegenerative diseases, particularly PD, because of the 8-hydroxyquinoline and catecholamine moieties in its chemical structure (Figure 1). Both moieties are antioxidative and metal-chelating scaffolds that have been linked to anti-neurodegenerative effects [10,11].

Recently, we intensively studied the synthesis of pyridine-fused privileged scaffolds and their applications employing Ag(I)-catalyzed cycloisomerization of *N*-aryl propargylamine precursors. Taking advantage of the feasibility of pyridine construction from acid-labile precursors, we synthesized the acid-labile lactone-fused pyridine scaffolds such as pyridocoumarines and pyranoquinolines [12,13,14]. The successful applications of Ag(I)-catalyzed cycloisomerization and interesting structural features of **1** prompted us to attempt a total synthesis and investigate the biological activities of **1**. Herein, we describe the total synthesis of the proposed structure of **1** and its protective properties against DA neurodegeneration in the *C. elegans* disease model system.

## 2. Results and Discussion

**Figure 2:**
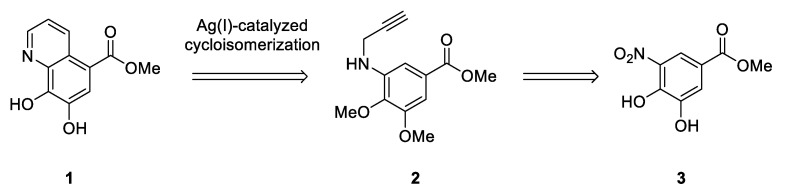
Retrosynthetic analysis for aaptoline A (**1**).

The retrosynthetic analysis of **1** (Figure 2) includes a high-yielding regioselective Ag(I)-catalyzed cycloisomerization. We anticipated that **1** could be obtained from dimethoxy-propargylaminobenzoate **2**, which contains the carbon skeleton of the target compound, by cycloisomerization and subsequent *O*-demethylation. We expected that **2** could be synthesized from known dihydroxynitrobenzoate **3** via nitro reduction and subsequent *N*-propargylation.

**Scheme 1:**
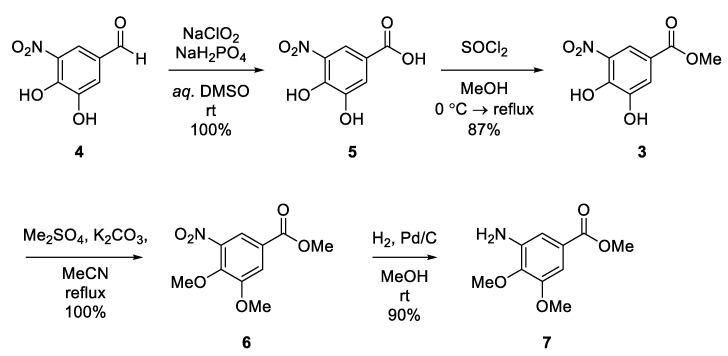
Synthesis of dimethoxyaniline intermediate **7**.

As shown in Scheme 1, our synthesis commenced with the preparation of amino-dimethoxybenzoate 7, a key intermediate for quinoline skeleton construction, according to literature procedures with some modifications [15,16,17]. Benzaldehyde 3 was stirred with sodium chlorite and sodium phosphate monobasic (NaH_2_PO_4_) to obtain dihydroxybenzoic acid 5 [15], which was subjected to an esterification reaction using thionyl chloride to provide methyl benzoate 3 [16]. Etherification with dimethyl sulfate was performed to protect the nucleophilic catechol moiety of 3, and known dimethoxybenzoate 6 [17] was prepared in quantitative yield. Aminobenzoate 7 was readily synthesized from nitrobenzoate 6 by hydrogenation in the presence of a catalytic amount of palladium on activated carbon.

**Scheme 2:**
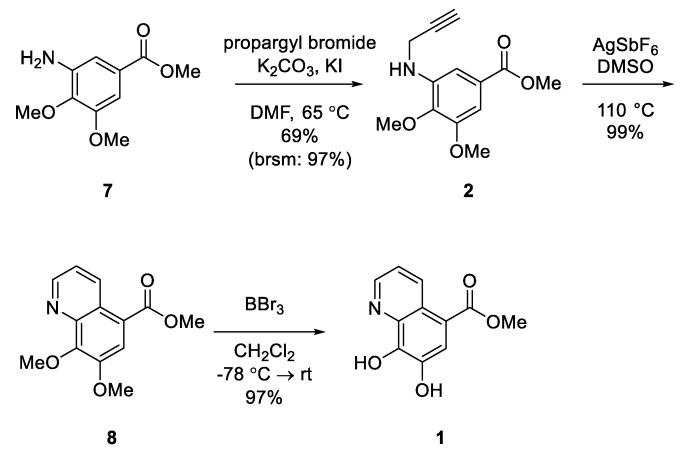
Completion of the synthesis of the proposed structure of aaptoline A (**1**).

With aniline **7** in hand, we carried out the *N*-propargylation to provide *N*-aryl propargylamine **2**, a key intermediate for Ag(I)-catalyzed cycloisomerization as shown in Scheme 2. To minimize the dipropargylated byproducts, we intensively investigated the reaction conditions. Without the dipropargylated byproduct, we obtained **2** in 69% yield along with reusable starting material (97% yield brsm.) by heating a DMF solution of **7** and one equivalent of propargyl bromide, 2.2 equivalents of K_2_CO_3_, and a catalytic amount of KI at 65 °C for 24 h. Cycloisomerization of **2** using a catalytic amount of AgSbF_6_ [12,18] afforded 7,8-dimethoxyquinoline **8** in high yield (99%). Demethylation of **8** using boron tribromide and subsequent reverse-phase column chromatography afforded the desired dihydroxyquinoline **1** in a high isolated yield (97%).

**Figure 3:**
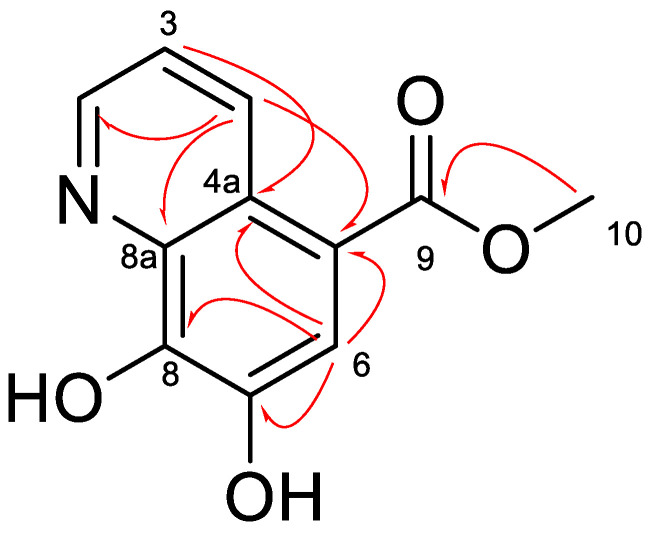
Selected HMBC correlations (H→C) of synthesized **1**.

Unfortunately, the NMR spectra of **1** differed from the reported data when using the same measurement conditions (500 MHz, CD_3_OD). Significant differences were observed between the ^1^H and ^13^C NMR spectra of our synthetic sample and those of the natural product [8]. Given that the substituents, including the two phenols and methyl carboxylate moiety, originated from commercially available dihydroxynitrobenzaldehyde **4**, the location of these three substituents could be confirmed, and we assigned the chemical shifts of synthesized **1** with a focus on the newly formed pyridine moiety. The structure of synthesized **1** was confirmed by combined 2D-NMR analysis (see Supplementary Materials). The protons at C-3 and C-6 were clearly identified via homonuclear correlation spectroscopy (^1^H-^1^H COSY) correlations with neighboring aromatic protons at C-2 and C-4, which were confirmed by heteronuclear single quantum coherence (HSQC) and heteronuclear multiple bond correlation (HMBC) studies. Carbons-bearing hydrogens, such as C-3 and C-6, were unambiguously assigned using HSQC analysis. The ^13^C-NMR spectral data for the quaternary carbons (C-4a, 8a, and 5) were carefully assigned by employing the HMBC correlations. Overall, our assignment of **1** seems to be more reasonable compared to the previous report. Selected HMBC correlations and their assignments are shown in Figure 3 and Table S1, respectively.

**Figure 4:**
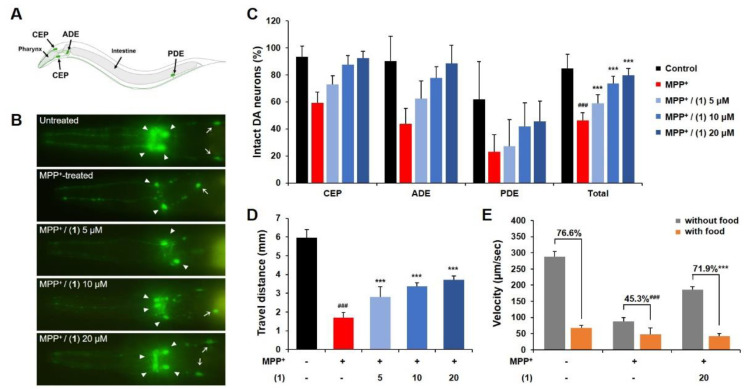
Protective effects of synthesized **1** on the MPP^+^-induced DA neurodegeneration in *C. elegans*. (**A**) DA neuronal circuit of *C. elegans*. (**B**) GFP expression patterns of CEP (arrowhead) and ADE (arrow) in the transgenic strain BZ555 (Pdat-1::GFP) were photographed at 400× magnification using a fluorescence microscope. (**C**) The viability of all eight DA neurons was scored by inspecting the fluorescence signals of BZ555 worms. (**D**) The movement of individual wild-type worms was tracked for 20 s, and the travel distance was recorded by an automated behavior tracking system under a dissecting microscope. (**E**) The basal slowing response was calculated by measuring the respective velocity of wild-type worms in the presence or absence of food. Statistical significance was determined by one-way ANOVA. ^###^ *p* < 0.001 compared with vehicle alone. *** *p* < 0.001 compared with MPP⁺-treated control. Error bars indicate standard deviation.

Considering the privileged moieties embedded in **1**, such as hydroxyquinoline and catecholamine, and the suggested biosynthetic pathway from dopamine [8], synthesized **1** was expected to have therapeutic properties against PD. Therefore, we evaluated the neuroprotective effect of synthesized **1** on DA neurodegeneration using the *C. elegans* model system. *C. elegans* has eight DA neurons, including four cephalic (CEP) neurons, two anterior deirid (ADE) neurons, and two posterior deirid (PDE) neurons (Figure 4A). In this study, the viability of all DA neurons was scored by observing fluorescent signals of transgenic worms expressing green fluorescent protein (GFP) in DA neurons to clarify the effect of synthesized **1** on MPP^+^-induced DA neuronal toxicity. The treatment of young adult worms with 4 mM of MPP^+^ for 96 h led to a marked decrease in the number of intact DA neurons, validating the capability of MPP^+^ to impair DA neurons. In contrast to the vehicle-treated worms, MPP^+^-induced reduction in DA neuronal viability was significantly recovered in the **1**-fed worms in a dose-dependent manner (Figure 4B,C). As demonstrated in Figure 4B, treatment with **1** remarkably restored the MPP^+^-induced morphological changes in DA neurons, including dendrite blebbing, cell body rounding, and loss.

To address whether this **1**-mediated DA neuroprotection was sufficient to influence DA neuron-related functional characteristics, MPP^+^-treated wild-type worms were subjected to behavioral assays. Since MPP^+^ exposure can alter the locomotor ability of worms that express PD symptoms, the travel distance test was employed to determine the effect of **1** on the motility defects of MPP^+^-exposed worms. In this study, **1** was found to reverse the decreased locomotor ability of MPP^+^-exposed worms in a dose-dependent manner (Figure 4D). The worms on the bacterial lawn move slowly to consume food. This basal slowing response of worms is regulated by the DA neuronal circuit [19]. As shown in Figure 4E, MPP^+^ exposure to worms caused a significant reduction in the slowing rate of approximately 40%, compared to that of vehicle-treated worms. When treated with 20 µM of **1**, the food-sensing performance of worms dramatically improved by up to 93.7% of that of vehicle-treated worms (Figure 4E). Together, our results demonstrate that MPP^+^-induced DA neuronal death and consequent DA neuron-mediated sensing and motor deficits of worms could be rescued by **1**, indicating its ability to provide both morphological and functional DA neuroprotection in *C. elegans*. In conclusion, **1** may be a suitable candidate in the search for novel drugs to combat PD.

## 3. Materials and Methods

### 3.1. Chemistry

#### 3.1.1. General Experimental

Unless otherwise noted, all reactions were performed under an argon atmosphere in oven-dried glassware. The starting materials and solvents were used as received from commercial suppliers without further purification. Thin-layer chromatography was carried out using Merck silica gel 60 F254 plates and visualized with a combination of UV, *p*-anisaldehyde, and potassium permanganate staining. Flash chromatography was performed using Merck silica gel 60 (0.040–0.063 mm, 230–400 mesh). Mass spectra were obtained using an Agilent 6530 Q-TOF unit. ^1^H and ^13^C spectra were recorded on a Bruker 500’54 Ascend spectrometer operating at 500 MHz at the Center for Bio-Medical Engineering Core Facility (Dankook University, Cheonan, Chungnam, Korea) in deuterated solvents. ^1^H and ^13^C NMR chemical shifts are reported in parts per million (ppm) relative to TMS, with the residual solvent peak used as an internal reference. Signals are reported as m (multiplet), s (singlet), d (doublet), t (triplet), q (quartet), bs (broad singlet), bd (broad doublet), dd (doublet of doublets), dt (doublet of triplets), or dq (doublet of quartets); the coupling constants are reported in hertz (Hz).

#### 3.1.2. 3,4-Dihydroxy-5-nitrobenzoic Acid (**5**)

Benzoic acid **5** was obtained from **4** according to literature procedures with some modifications [15]. Briefly, to a solution of 3,4-dihydroxy-5-nitrobenzaldehyde **4** (1.00 g, 5.46 mmol) and sodium dihydrogen phosphate (938 mg, 6.01 mol) in H_2_O (2.2 mL) and DMSO (5.5 mL), a solution of sodium chlorite (975 mg, 10.78 mmol) in H_2_O (5.1 mL) was added dropwise for 90 min at ambient temperature. After being stirred at the same temperature for an additional 30 min, the reaction mixture was poured into a 5% *aq*. NaHCO_3_ and CH_2_Cl_2_. The aqueous layer was acidified with conc. HCl and extracted with diethyl ether. The organic layer was washed with brine, dried over MgSO_4_, and evaporated to afford the benzoic acid **5** (1.08 g, quant.) as a yellow solid. ^1^H-NMR (500 MHz, CD_3_OD) δ 8.19 (d, 1H, *J* = 2.0 Hz), 7.68 (d, 1H, *J* = 1.9 Hz); ^13^C-NMR (125 MHz, CD_3_OD) δ 166.5, 147.7, 146.7, 134.8, 121.5, 120.3, 117.0.

#### 3.1.3. Methyl 3,4-Dihydroxy-5-nitrobenzoate (**3**)

Methylbenzoate **3** was obtained from **5** according to literature procedures with some modifications [16]. Briefly, to a solution of benzoic acid **5** (1.46 g, 7.32 mmol) in MeOH (29 mL) was added dropwise thionyl chloride (1.2 mL, 16.84 mmol) at 0 °C. The reaction mixture was refluxed for 150 min and concentrated. The residue was solidified from water and dried *in vacuo* to afforded methyl benzoate **3** (1.35 g, 87%) as a yellow solid. ^1^H-NMR (500 MHz, CD_3_OD) δ 8.09 (d, 1H, *J* = 1.7 Hz), 7.58 (d, 1H, *J* = 1.8 Hz), 3,82 (s, 3H); ^13^C-NMR (125 MHz, CD_3_OD) δ 166.7, 149.3, 148.2, 136.3, 122.2, 121.3, 118.2, 52.9.

#### 3.1.4. Methyl 3,4-Dimethoxy-5-nitrobenzoate (**6**)

Dimethoxybenzoate **6** was obtained from **3** according to literature procedures with some modifications [17]. Briefly, to a solution of dihydroxybenzoate **3** (230 mg, 1.08 mmol) and K_2_CO_3_ (747 mg, 5.4 mmol) in CH_3_CN (10 mL) was added Me_2_SO_4_ (682 mg, 5.3 mmol) at ambient temperature. The reaction mixture was refluxed for 4 h and cooled to ambient temperature. The insoluble material was filtered off and washed with CH_3_CN. The filtrate was concentrated in vacuo and purified via column chromatography on silica gel (EtOAc:*n*-hexane = 1:10–1:4) to afford **6** (260 mg, quant.) as white solid. ^1^H-NMR (500 MHz, CD_3_OD) δ 7.98 (d, 1H, *J* = 1.7 Hz), 7.89 (d, 1H, *J* = 1.4 Hz), 4.06 (d, 6H, *J* = 4.8 Hz), 3.99 (s, 3H); ^13^C-NMR (125 MHz, CD_3_OD) δ 166.3, 155.4, 147.3, 146.0, 127.0, 118.1, 117.4, 62.5, 57.2, 53.1.

#### 3.1.5. Methyl 3-Amino-4,5-dimethoxybenzoate (**7**)

A mixture of nitrobenzoate **6** (250 mg, 1.04 mmol) and catalytic amount of 10% Pd/C in MeOH (15 mL) was stirred under H_2_ atmosphere at ambient temperature for 6 h. The reaction mixture was filtered, and the filtrate was concentrated in vacuo. Purification of residue via column chromatography on silica gel (EtOAc:*n*-hexane = 1:6–1:3) afforded aminobenzoate **7** (196 mg, 90%) as a yellow oil. ^1^H-NMR (500 MHz, CD_3_OD) δ 7.19 (d, 1H, *J* = 1.8 Hz), 7.06 (d, 1H, *J* = 1.7 Hz), 3.91 (s, 6H), 3.89 (s, 3H); ^13^C-NMR (125 MHz, CD_3_OD) δ 168.8, 153.9, 142.5, 141.1, 126.8, 111.7, 104.2, 60.2, 56.3, 52.5; HR-MS (Q-ToF): Calcd for C_10_H_14_NO_4_^+^ (M + H^+^): 212.0917. Found: 212.0919.

#### 3.1.6. Methyl 3,4-Dimethoxy-5-(prop-2-yn-1-ylamino)benzoate (**2**)

To a solution of **7** (986 mg, 4.67 mmol), KI (78 mg, 0.47 mmol) and K_2_CO_3_ (1.42 g, 10.27 mmol) in DMF (15 mL) was added 695 mg (4.67 mmol) of 80 *w*/*w*% solution of propargyl bromide in toluene at ambient temperature. The reaction mixture was stirred for 24 h at 65 °C, cooled to ambient temperature, and then diluted with water and EtOAc. The organic layer was washed with water and brine, dried over MgSO_4_, and concentrated in vacuo. Purification of residue via column chromatography on silica gel (EtOAc:*n*-hexane = 1:10–1 3) afforded *N*-propargyl aminobenzoate **2** (805 mg, 69%) as a yellowish oil along with 280 mg of starting material **2** (97% yield brsm). ^1^H-NMR (500 MHz, CD_3_OD) δ 7.21 (d, 1H, *J* = 2.1 Hz), 7.15 (d, 1H, *J* = 1.9 Hz), 4.04 (d, 2H, *J* = 2.0 Hz), 3.93 (d, 6H, *J* = 6.2 Hz), 3.88 (s, 3H), 2.56 (s, 1H); ^13^C-NMR (125 MHz, CD_3_OD) δ 168.9, 153.4, 142.6, 141.4, 126.8, 107.8, 104.7, 82.2, 71.9, 60.5, 56.4, 52.6, 33.6; HR-MS (Q-ToF): Calcd for C_13_H_16_NO_4_^+^ (M + H^+^): 250.2735. Found: 250.2738.

#### 3.1.7. Methyl 7,8-Dimethoxyquinoline-5-carboxylate (**8**)

To a solution of **2** (10 mg, 0.04 mmol) in DMSO (1 mL) was added AgSbF_6_ (1.4 mg, 4 μmol) at ambient temperature. The reaction mixture was stirred at 110 °C for 8.5 h, cooled to ambient temperature, diluted with EtOAc and quenched with saturated *aq*. NaHCO_3_ solution. The organic layer was washed with water and brine, dried over Na_2_SO_4_, and concentrated in vacuo. Purification of residue via column chromatography on silica gel (EtOAc:*n*-Hexane = 1:7) afforded quinoline **8** (9.8 mg, 99%) as a yellow oil. ^1^H-NMR (500 MHz, CD_3_OD) δ 9.34 (d, 1H, *J* = 8.8 Hz), 8.82 (d, 1H, *J* = 2.3 Hz), 8.21 (s, 1H), 7.49 (1H, dd, *J* = 8.7, 4.2 Hz), 4.05 (6H, d, *J* = 12.3 Hz), 3.97 (s, 3H); ^13^C-NMR (125 MHz, CD_3_OD) δ 167.6, 151.4, 151.4, 148.1, 143.8, 136.3, 124.8, 123.0, 122.1, 121.5, 62.1, 57.5, 52.8; HR-MS (Q-ToF): Calcd for C_13_H_14_NO_4_^+^ (M + H^+^): 248.0917. Found: 248.0917.

#### 3.1.8. Aaptoline A (**1**)

To a solution of dimethoxyquinoline **8** (26.5 mg, 0.107 mmol) in CH_2_Cl_2_ (1 mL) was added slowly 1 M BBr_3_ in CH_2_Cl_2_ (0.3 mL, 0.3 mmol) at −78 °C. The reaction mixture was stirred at same temperature for 1 h and then at ambient temperature overnight. The reaction mixture was quenched with water and diluted with CH_2_Cl_2_. The organic layer was washed with water and brine, dried over Na_2_SO_4_, and concentrated in vacuo. Purification of residue via reverse-phase column chromatography using Biotage^®^ Select Purification system (Biotage^®^ Sfär-C-18, MeOH:H_2_O = 1:9–1:4, 6 mL/min) afforded **1** (22.8 mg, 97%) as an orange solid. ^1^H-NMR (500 MH, CD_3_OD) δ 9.38 (d, 1H, *J* = 7.5 Hz), 8.79 (s, 1H), 8.07 (s, 1H), 7.45 (d, 1H, *J* = 5.5 Hz), 3.96 (s, 3H); ^13^C-NMR (125 MHz, CD_3_OD) δ 168.1, 149.6, 144.6, 142.4, 140.2, 135.9, 125.3, 124.0, 121.5, 117.0, 52.4; HR-MS (Q-ToF): Calcd for C_11_H_10_NO_4_^+^ (M + H^+^): 220.0604. Found: 220.0608.

### 3.2. Biology

#### 3.2.1. *C. elegans* Incubation

In this study, wild-type N2 and transgenic strain: BZ555 (Pdat-1::GFP) were used. Both strains were obtained from the Caenorhabditis Genetic Center (CGC; University of Minnesota, Minneapolis, MN). The worms were grown in liquid medium and maintained at 20 °C. Live *E. coli* bacteria (OP50) was added to the liquid medium for providing the food source. For the MPP^+^ treatment, worms (young-adult stage) were incubated with or without **1**. After 2 hours of incubation, worms were exposed to MPP^+^ (4 mM) and incubated for another 96 h.

#### 3.2.2. Fluorescence Microscopy

On the fourth day of adulthood, BZ555 worms were immobilized with 10% sodium azide and mounted onto a 2% agarose pad. The DA neurodegeneration was microscopically investigated using a fluorescence microscope (Nikon Eclipse Ni-u, Tokyo, Japan). The number of normal DA neurons was counted in each worm by inspecting the GFP fluorescence signals. The fluorescence signals were quantified in each worm with image J software. A total of 25–30 worms from each group were analyzed in three independent experiments.

#### 3.2.3. Behavioral Assay

The effects of **1** on the locomotion and basal slowing response were investigated as described previously [20]. Briefly, to check the motor abilities, N2 worms were transferred to a fresh NGM plate, and their movements were tracked for 20 s under a dissecting microscope (Nikon SMZ1500, Tokyo, Japan) with Nikon image analysis software (NIS-Elements, Tokyo, Japan). For the food-sensing assay, well-fed N2 worms were transferred to a non-food plate and washed three times in M9 buffer to get rid of the remaining food. Then, the velocity of worms was calculated on the food-coated plate and non-coated plated, respectively.

#### 3.2.4. Statistical Analysis

All data presented were expressed as mean ± standard deviation as indicated. Statistical analysis was carried out using SPSS software. The calculation of statistical significance of differences among groups was conducted employing one-way analysis of variance (ANOVA) followed by Tukey test.

## 4. Conclusions

In summary, we have accomplished the total synthesis of the proposed structure of aaptoline A in seven steps from commercially available benzaldehyde **4** (52% overall yield). The key feature of our synthesis is the regioselective and high-yielding Ag(I)-catalyzed cycloisomerization of *N*-propargylaniline precursor **2** to construct a quinoline skeleton. The structure of **1** was confirmed by a combined 2D NMR analysis. Although the final compound did not match the NMR spectra of the natural product, synthetic methyl 7,8-dihydroxyquinoline-5-carboxylate (**1**), proposed as aaptoline A, was found to protect worms against MPP^+^-induced DA neurodegeneration, confirming its therapeutic potential against PD. Detailed mechanistic studies on its neuroprotective activity are underway and will be reported in due course.

## Data Availability

Not applicable.

## Supplementary Materials

The following are available online, Table S1: ^1^H- and ^13^C-NMR assignment of aaptoline A.
