# Supplementary material for: Synthesis of Proposed Structure of Aaptoline A, a Marine Sponge-Derived 7,8-Dihydroxyquinoline, and Its Neuroprotective Properties in C. elegans
Source: Molecules. 2021 Oct 1;26(19):5964. doi: 10.3390/molecules26195964 (PMC8512085; doi:10.3390/molecules26195964)

Supplementary Materials

Synthesis of proposed structure of aaptoline A, a marine sponge-derived 7,8-dihydroxyqunoline, and its neuroprotective properties in *C. elegans.*

Soobin Kim[1], Wooin Yang[2], Dong Seok Cha[2,*] and Young Taek Han[1,*]

[1]College of Pharmacy, Dankook University, Cheonan, Chungnam 31116, Korea;

[2]College of Pharmacy, Woosuk University, Wanju-gun, Jeonbuk 55338, Korea;

[*]Correspondence: hanyt@dankook.ac.kr (Y. T. Han); Tel.: +82-41-550-1431; cha@woosuk.ac.kr (D. S. Cha)

Content

Table S1. ^{1}H and ^{13}C-NMR assignment of aaptoline A -- S2

^{1}H NMR and ^{13}C NMR Spectra --- S3

2D NMR of aaptoline A (**1**)--- S16

Table S1. ^{1}H- and ^{13}C-NMR assignment of aaptoline A.

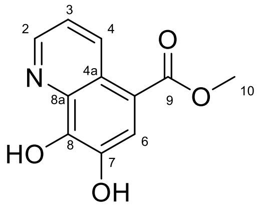

Position	Reported		Synthesized	
	δ_H (multi, *J* in Hz)	δ_C	δ_H (multi, *J* in Hz)	δ_C
2	8.92 (d, 4.6)	145.4	8.79 (s)	149.6
3	7.87 (dd, 4,6, 8.7)	119.6	7.45 (d, 5.5)	121.5
4	10.02 (d, 8.7)	144.1	9.38 (d, 7.5)	135.9
4a		124.6		124.0
5		119.1		117.0
6	8.28 (s)	126.8	8.07 (s)	125.3
7		147.4		142.4
8		139.2		144.6
8a		132.7		140.2
9		166.7		168.1
10	4.00 (s)	53.1	3.96 (s)	52.4

^{1}H NMR and ^{13}C spectra were recorded in CD_3OD at 500 MHz and 125 MHz, respectively.

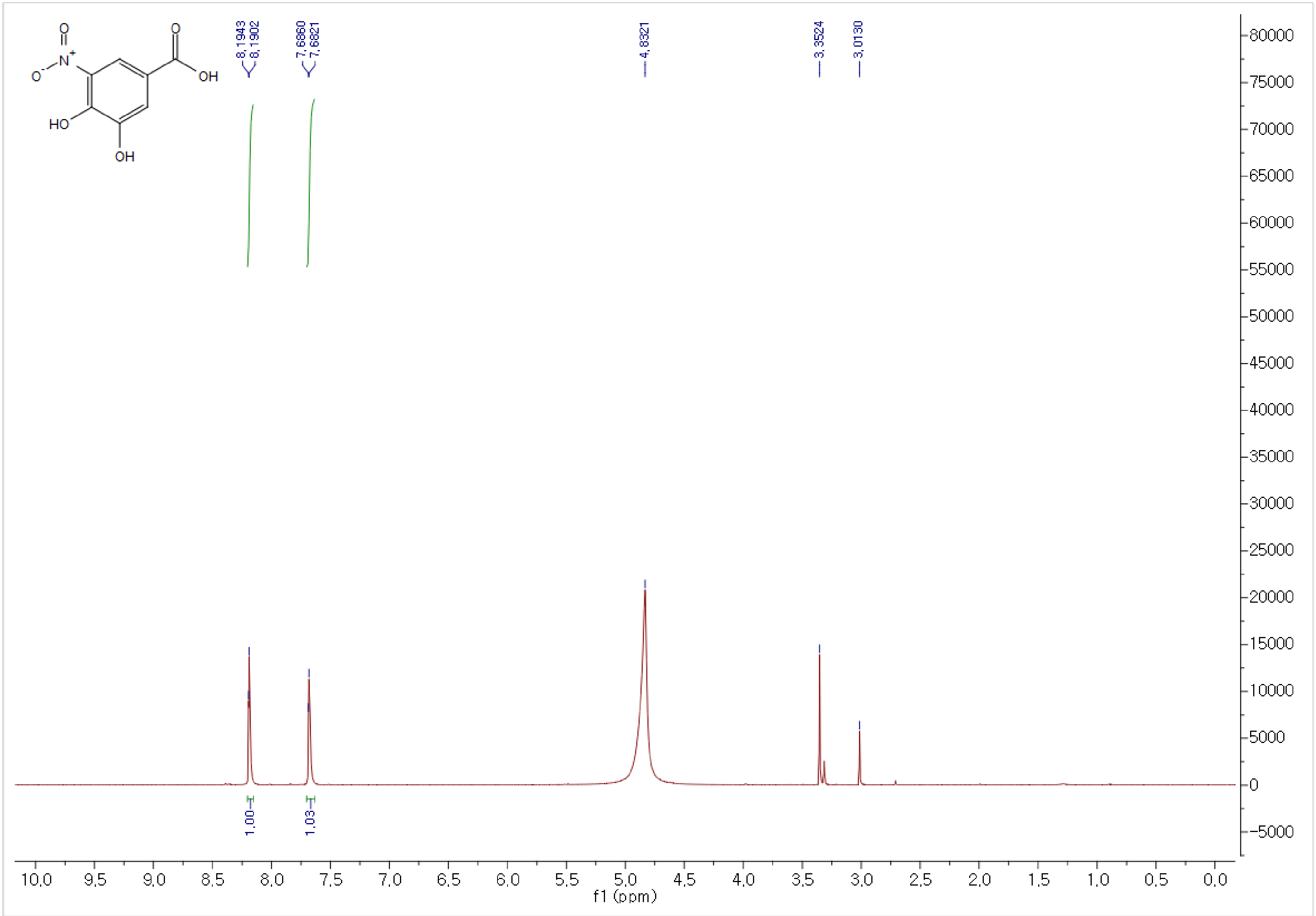

8.1943
8.1902
7.6860
7.6821
4.8321
3.3524
3.0130
1.00
1.03
f1 (ppm)

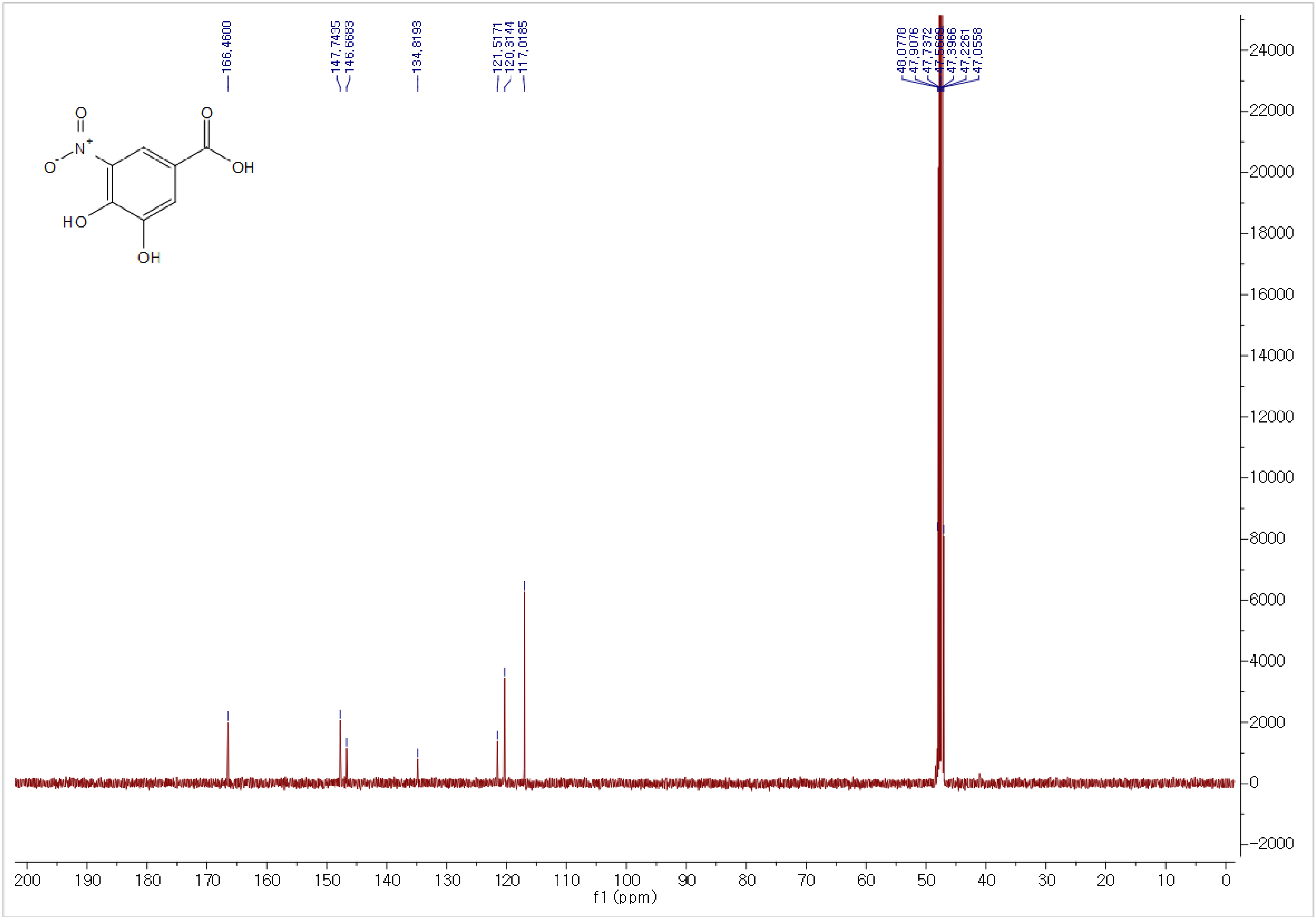

166.4600
147.7435
146.6683
134.8193
121.5171
120.3144
117.0185
48.0778
47.9076
47.7372
47.3966
47.2261
47.0558
OH
HO
OH
f1 (ppm)

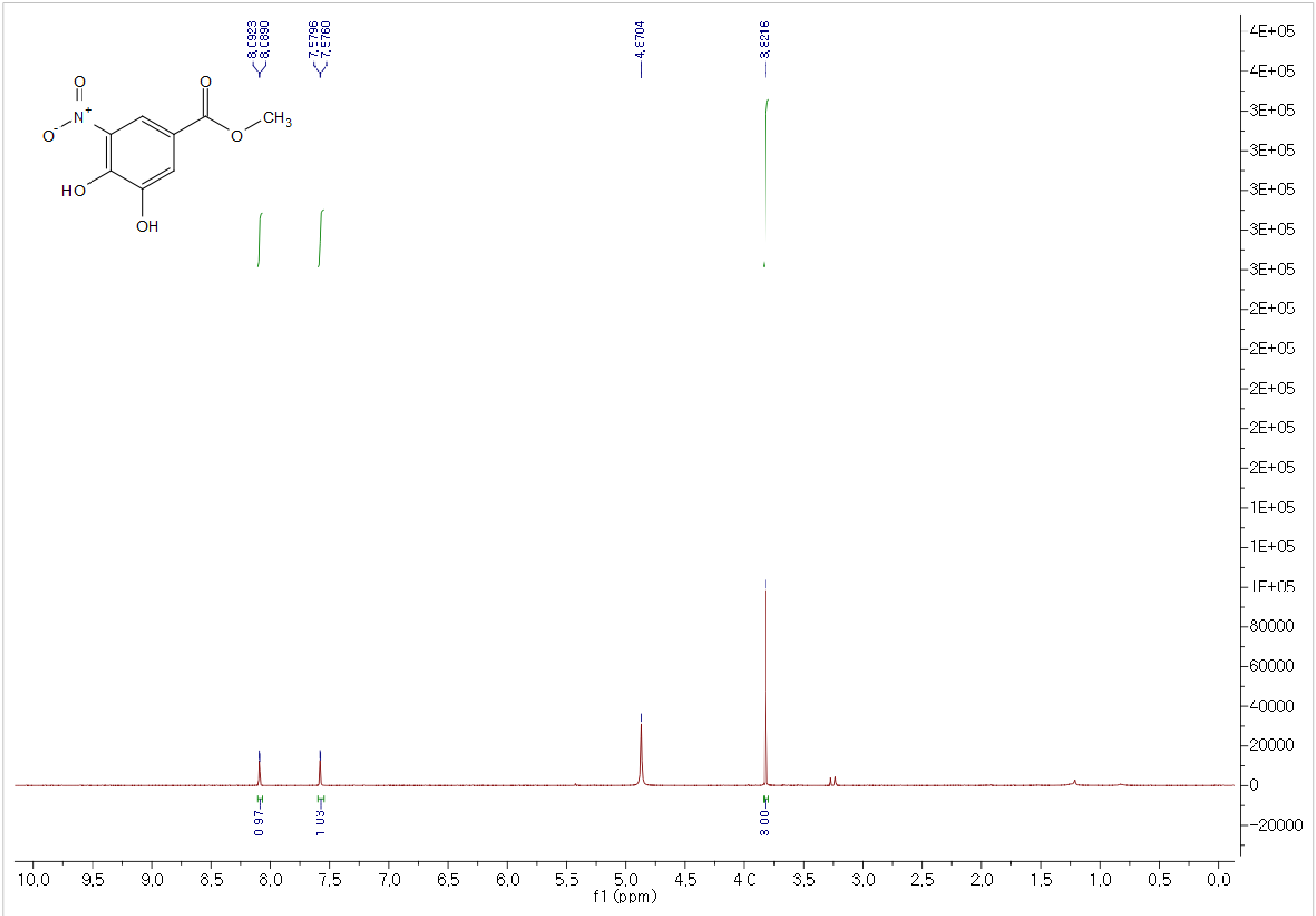

8.0923
8.0890
7.5796
7.5760
4.8704
3.8216
0.97
1.03
3.00
f1 (ppm)
CH3
HO
OH

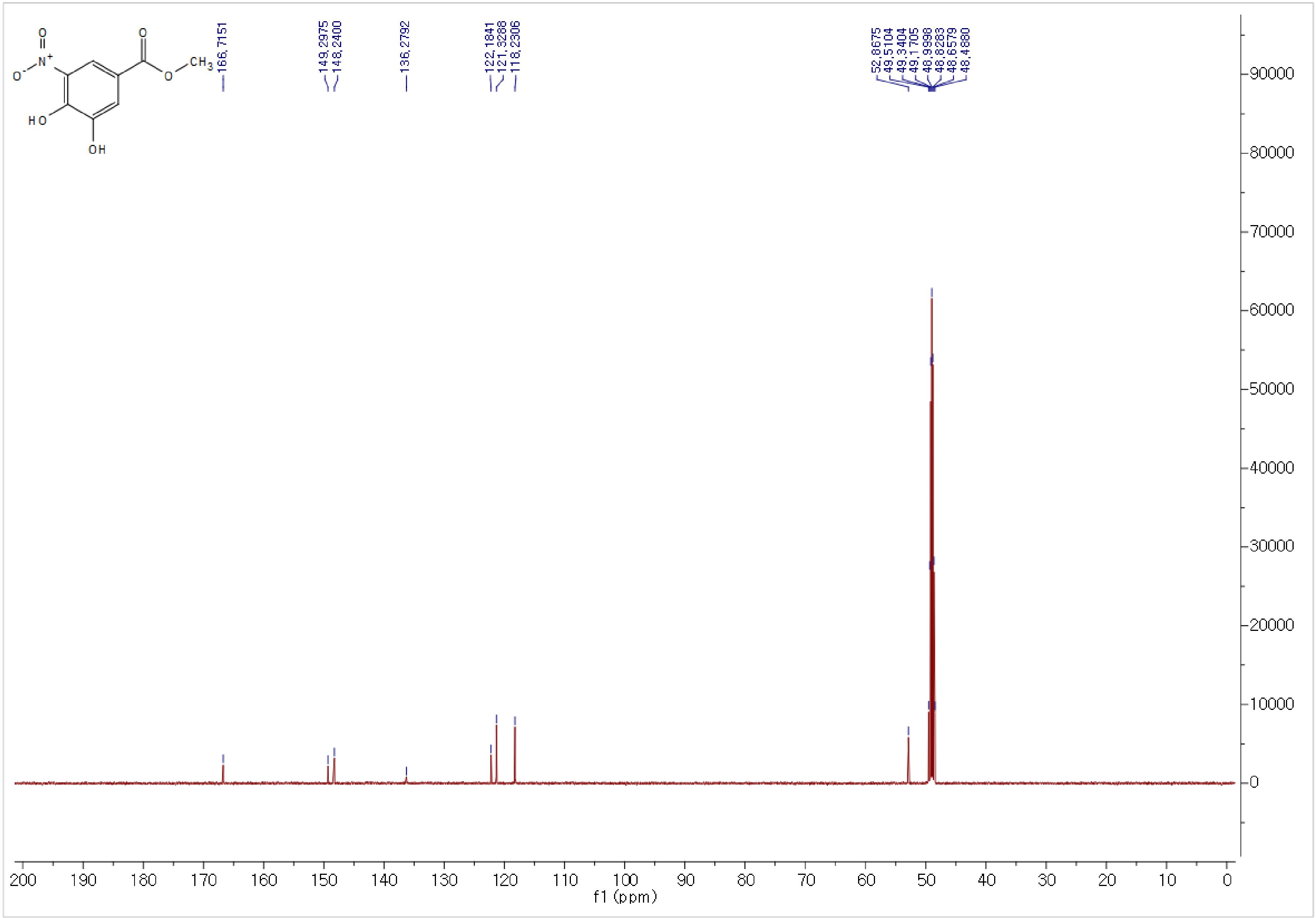

166.7151
149.2975
148.2400
136.2792
122.1841
121.3288
118.2306
52.8675
49.5104
49.3404
49.1705
48.9998
48.8283
48.6579
48.4880
f1 (ppm)

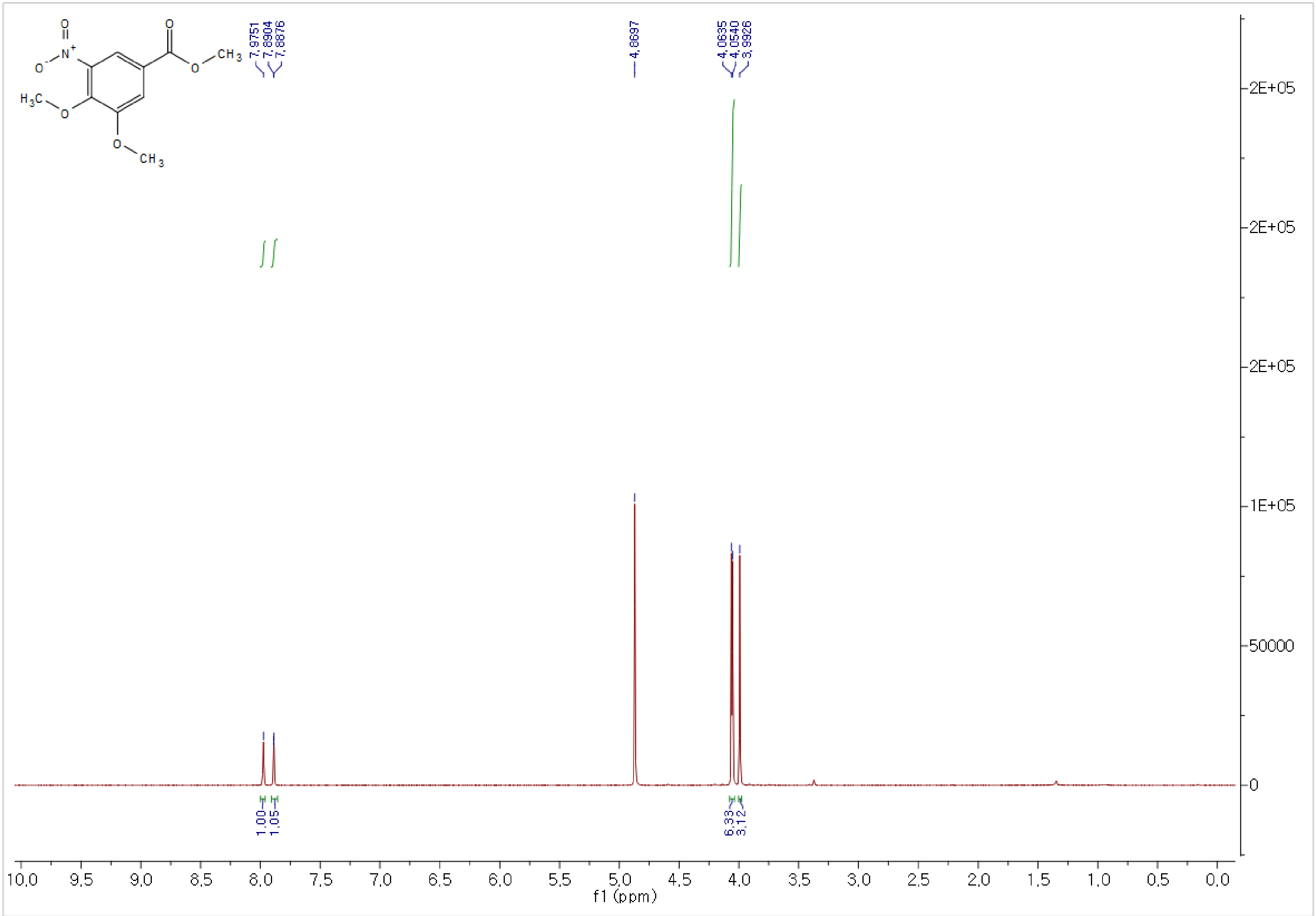

7.9751
7.8904
7.8876
4.8697
4.0635
4.0540
3.9926
1.00
1.05
6.33
3.12
2E+05
2E+05
2E+05
1E+05
50000
0
10.0 9.5 9.0 8.5 8.0 7.5 7.0 6.5 6.0 5.5 5.0 4.5 4.0 3.5 3.0 2.5 2.0 1.5 1.0 0.5 0.0
f1 (ppm)

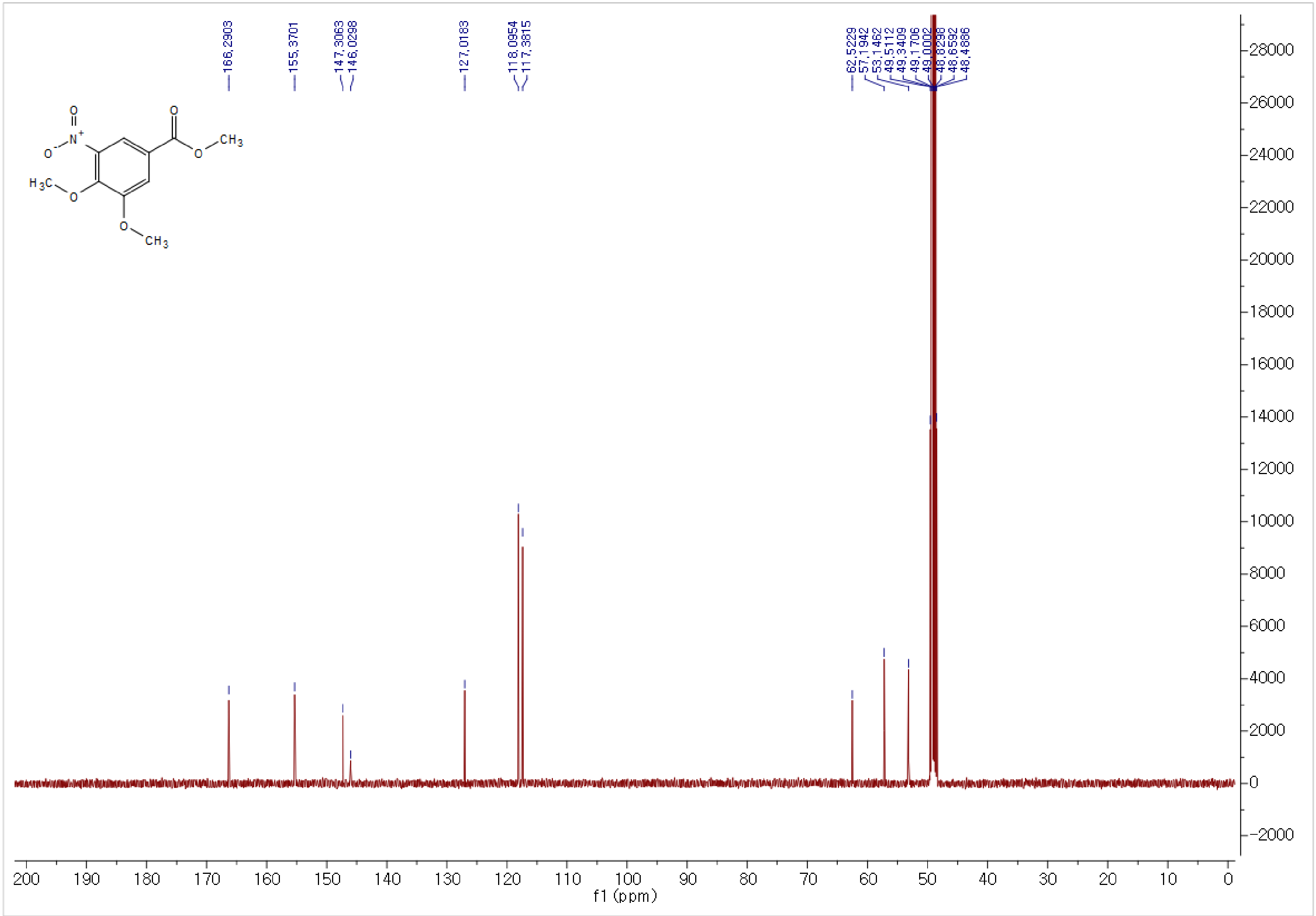

166.2903
155.3701
147.3063
146.0298
127.0183
118.0954
117.3815
62.5229
57.1942
53.1462
49.5112
49.3409
49.1706
49.0002
48.8298
48.6592
48.4886
f1 (ppm)

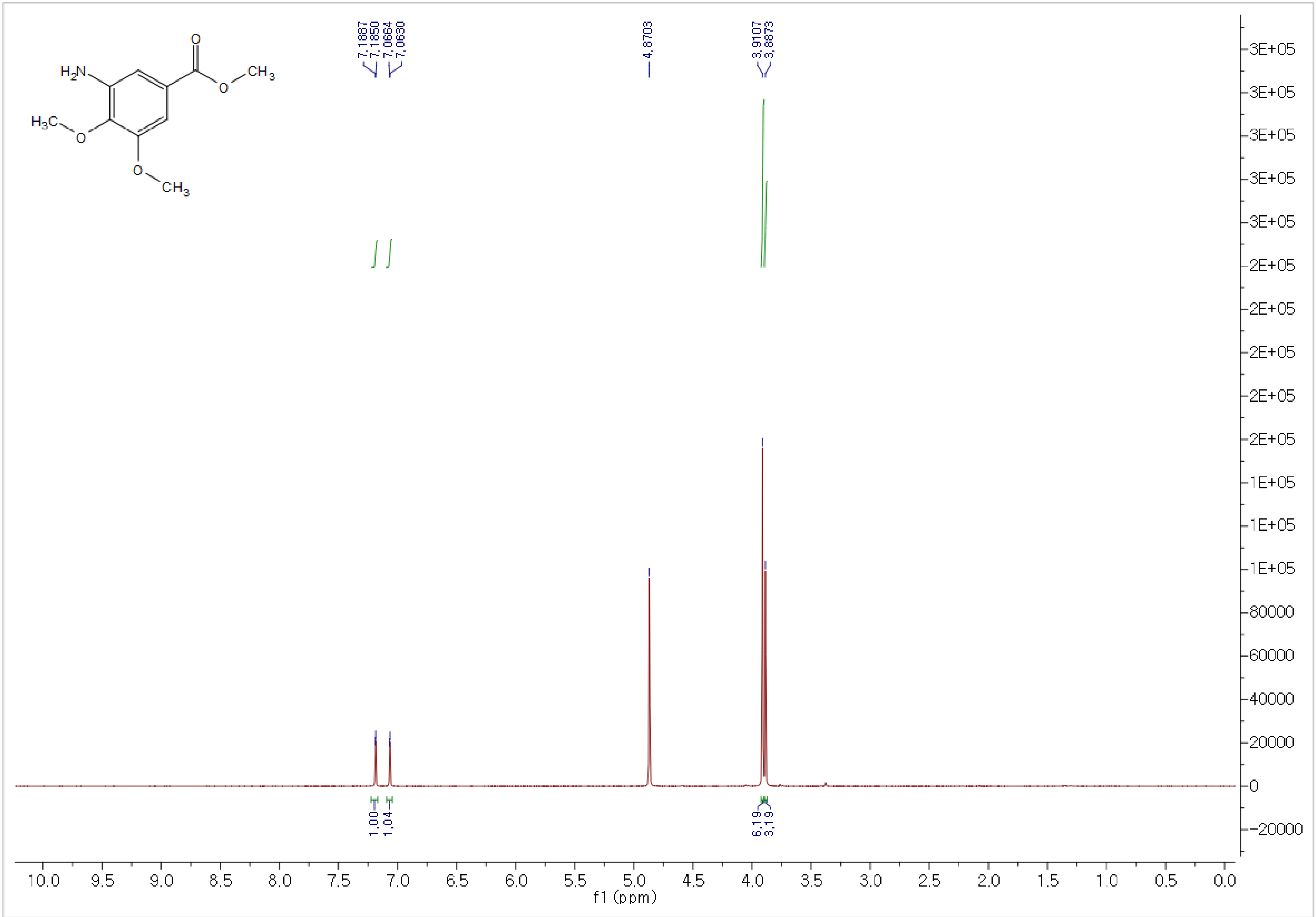

H2N
H3C
O
O
CH3
CH3
7.1887
7.1850
7.0664
7.0630
4.8703
3.9107
3.8873
1.00
1.04
6.19
3.19
f1 (ppm)

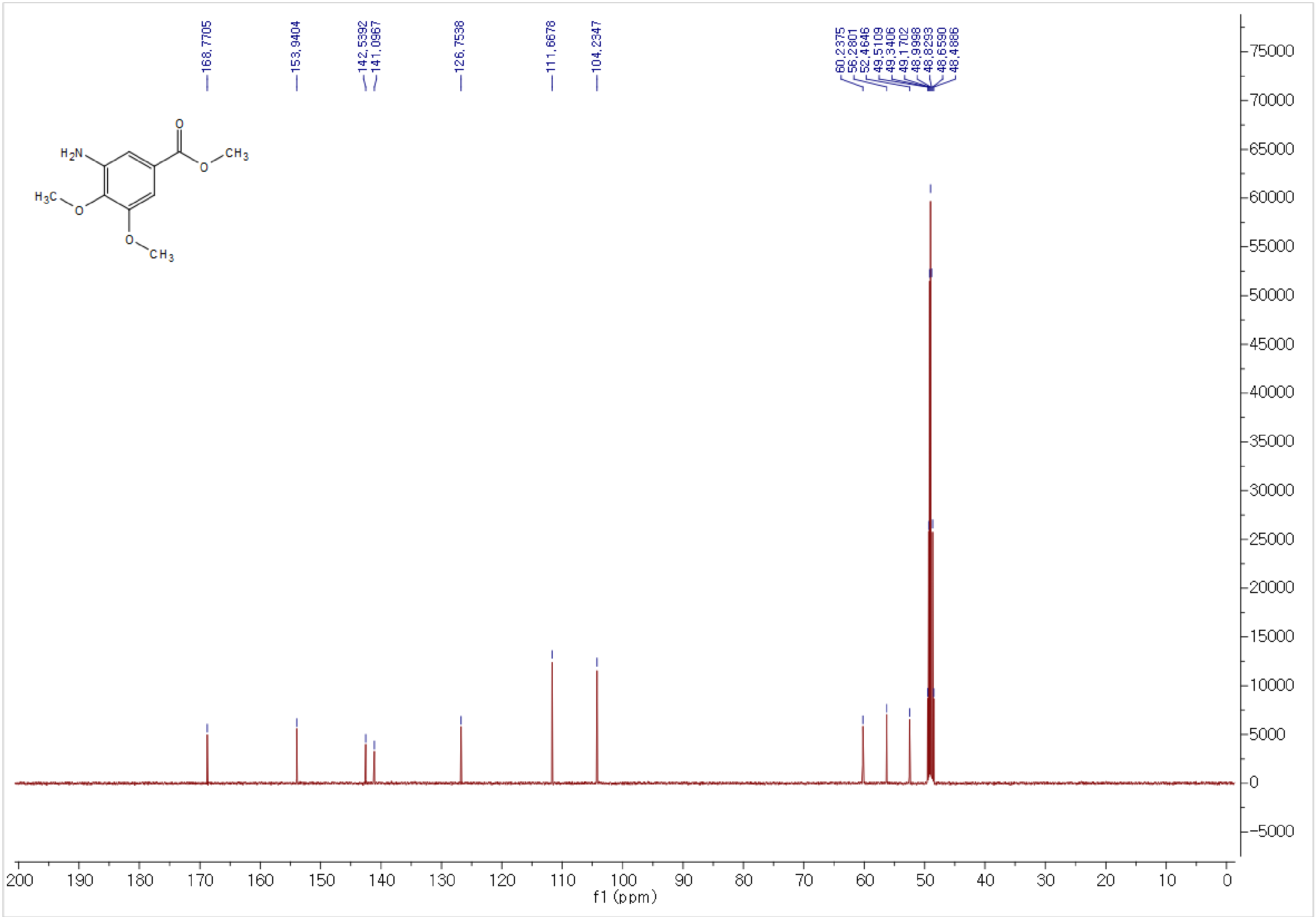

168.7705
153.9404
142.5392
141.0967
126.7538
111.6678
104.2347
60.2375
56.2801
52.4646
49.5109
49.3406
49.1702
48.9998
48.8293
48.6590
48.4886
f1 (ppm)

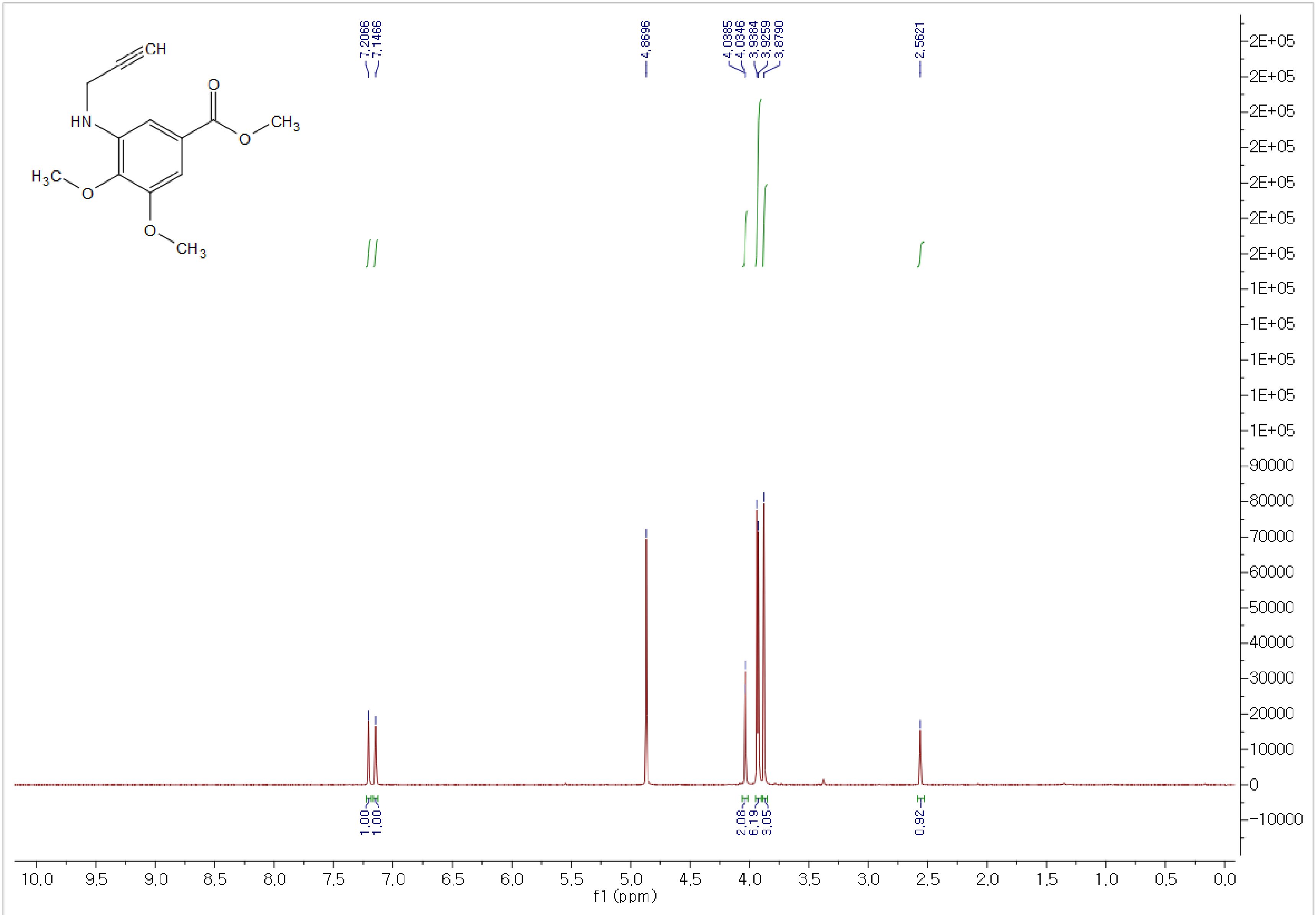

CH
O
HN
O
CH3
H3C
O
O
CH3
7.2066
7.1466
4.8696
4.0385
4.0346
3.9384
3.9259
3.8790
2.5621
1.00
1.00
2.08
6.19
3.05
0.92
f1 (ppm)

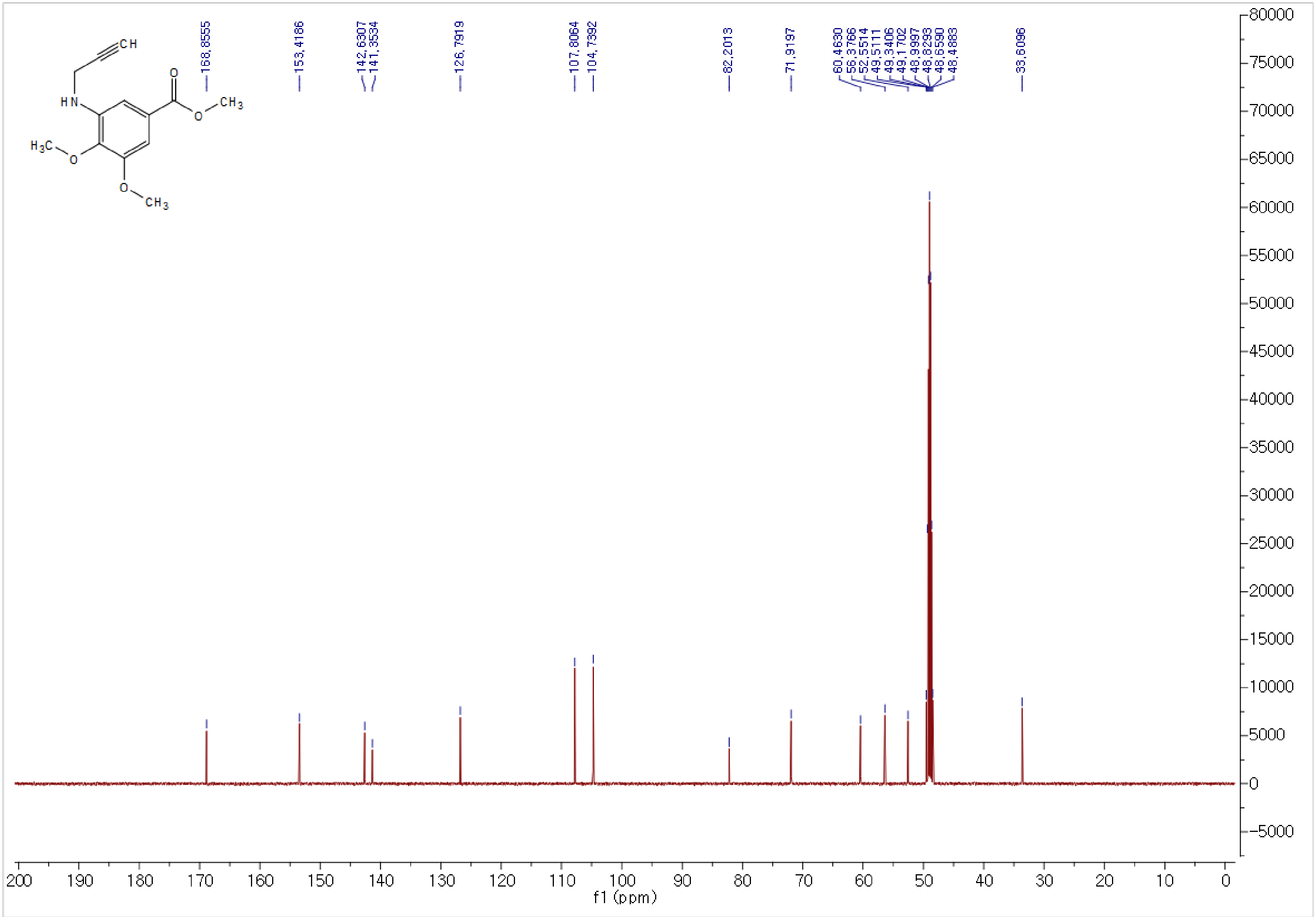

168,8555
153,4186
142,6307
141,3534
126,7919
107,8064
104,7392
82,2013
71,9197
60,4630
56,3766
52,5514
49,5111
49,3406
49,1702
48,9997
48,8293
48,6590
48,4883
33,6096
f1 (ppm)

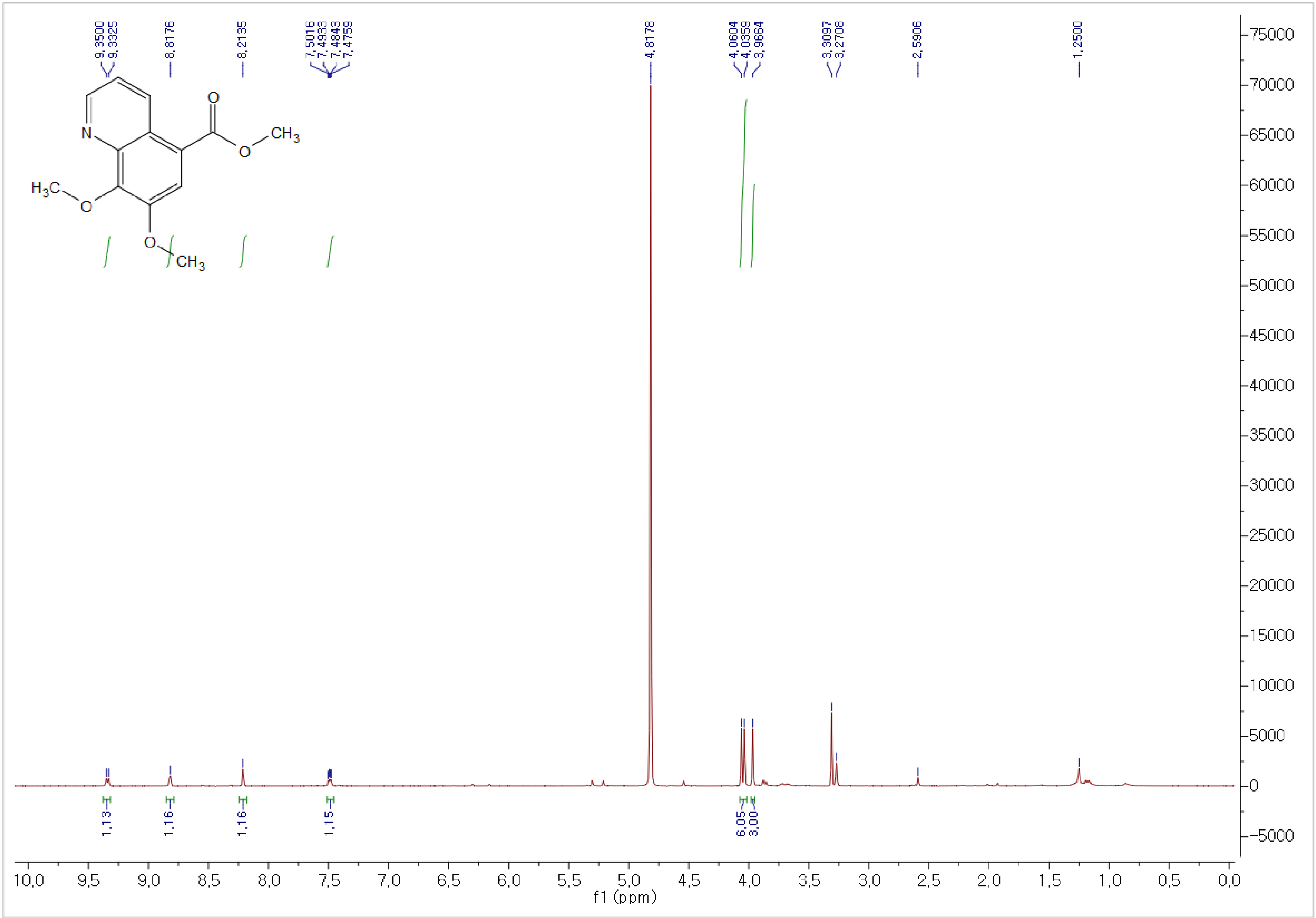

9,3500
9,3325
8,8176
8,2135
7,5016
7,4933
7,4843
7,4759
4,8178
4,0604
4,0359
3,9664
3,3097
3,2708
2,5906
1,2500
N
O
O
CH3
H3C
O
O
CH3
1,13
1,16
1,16
1,15
6,05
3,00
75000
70000
65000
60000
55000
50000
45000
40000
35000
30000
25000
20000
15000
10000
5000
0
-5000
10,0
9,5
9,0
8,5
8,0
7,5
7,0
6,5
6,0
5,5
5,0
4,5
4,0
3,5
3,0
2,5
2,0
1,5
1,0
0,5
0,0
f1 (ppm)

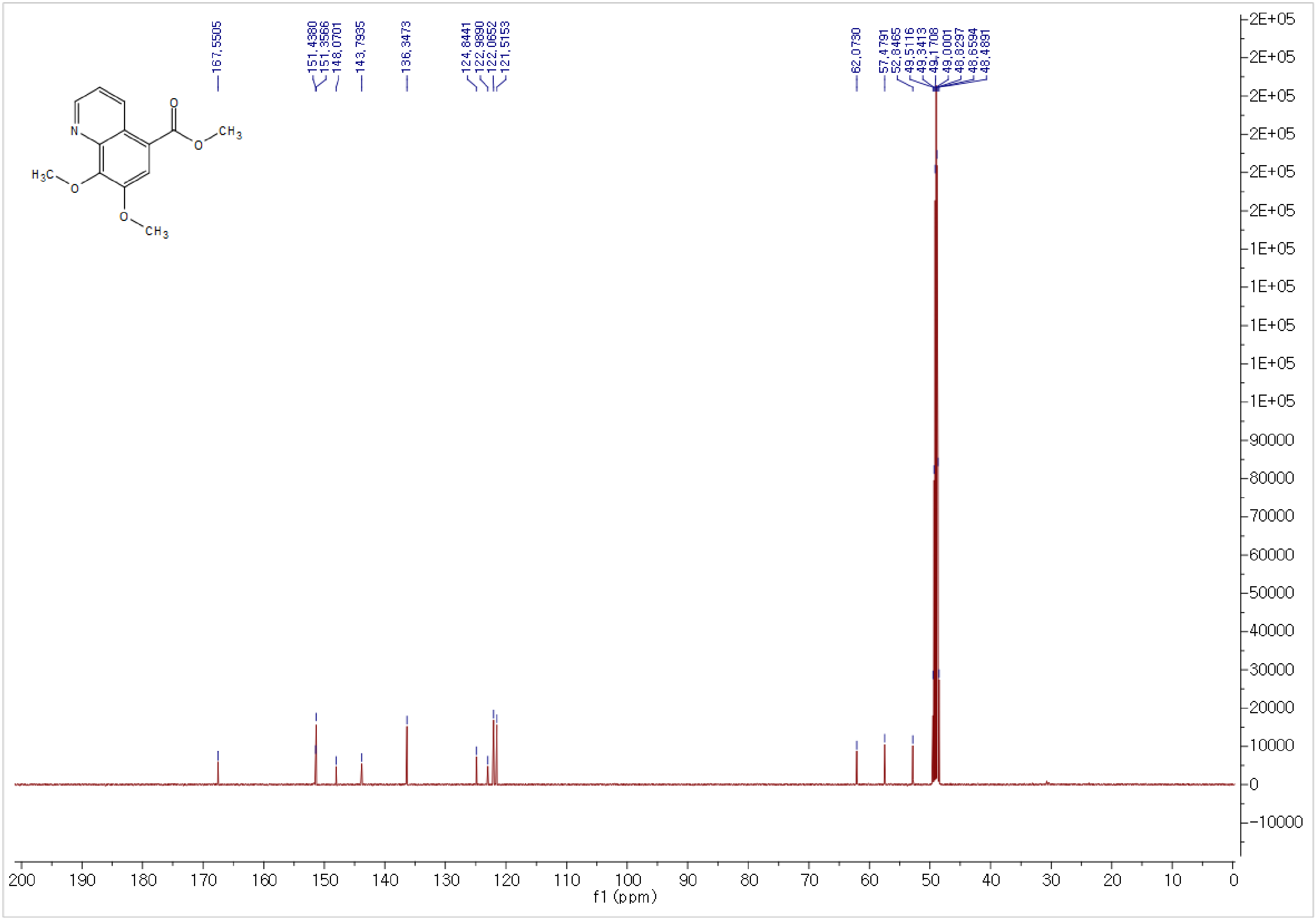

167.5505
151.4380
151.3566
148.0701
143.7935
136.3473
124.8441
122.9890
122.0652
121.5153
62.0730
57.4791
52.8465
49.5116
49.3413
49.1708
49.0001
48.8297
48.6594
48.4891
f1 (ppm)

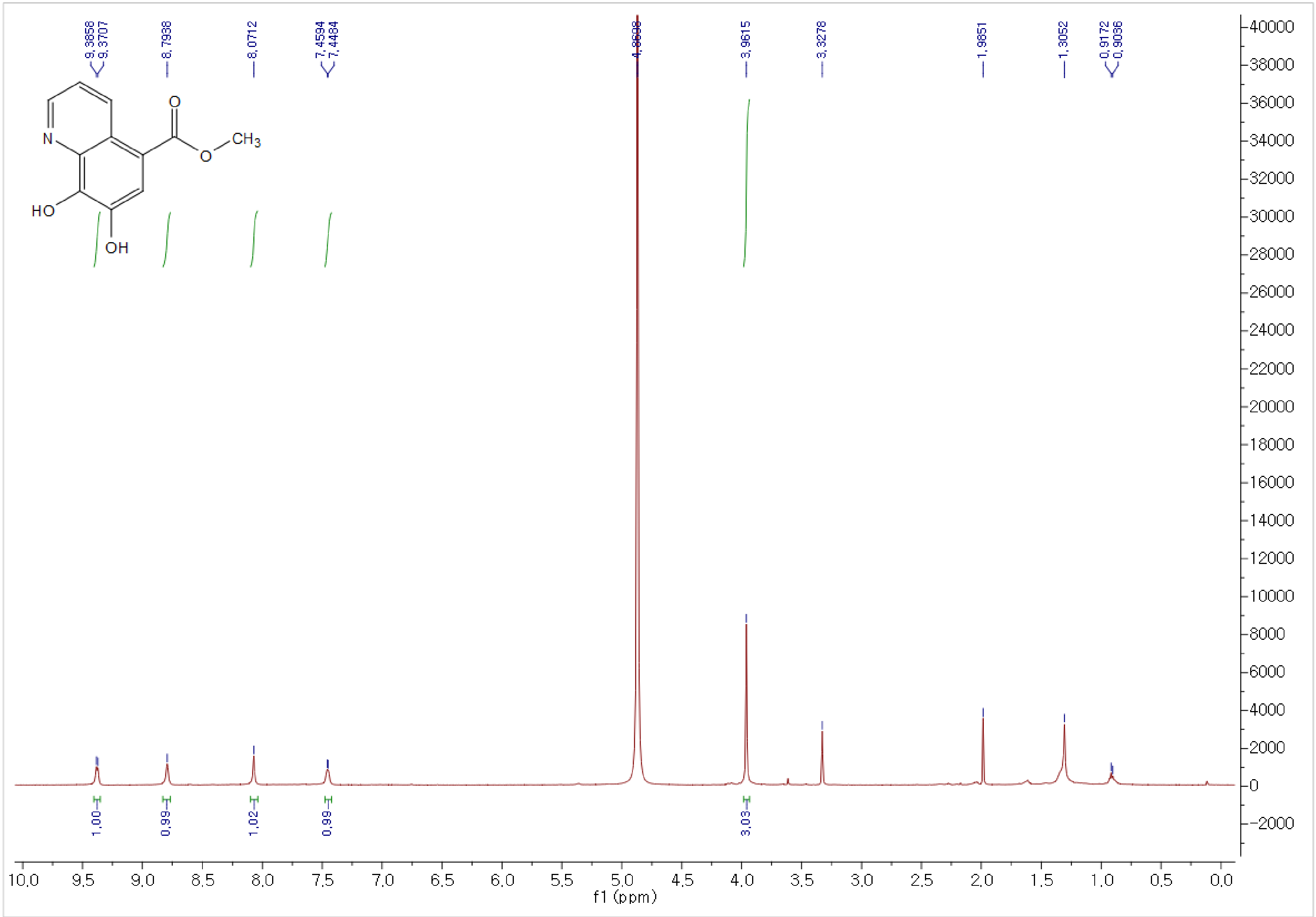

9.3858
9.3707
8.7938
8.0712
7.4594
7.4484
4.8698
3.9615
3.3278
1.9851
1.3052
0.9172
0.9036
N
O
O
CH3
HO
OH
1.00
0.99
1.02
0.99
3.03
f1 (ppm)

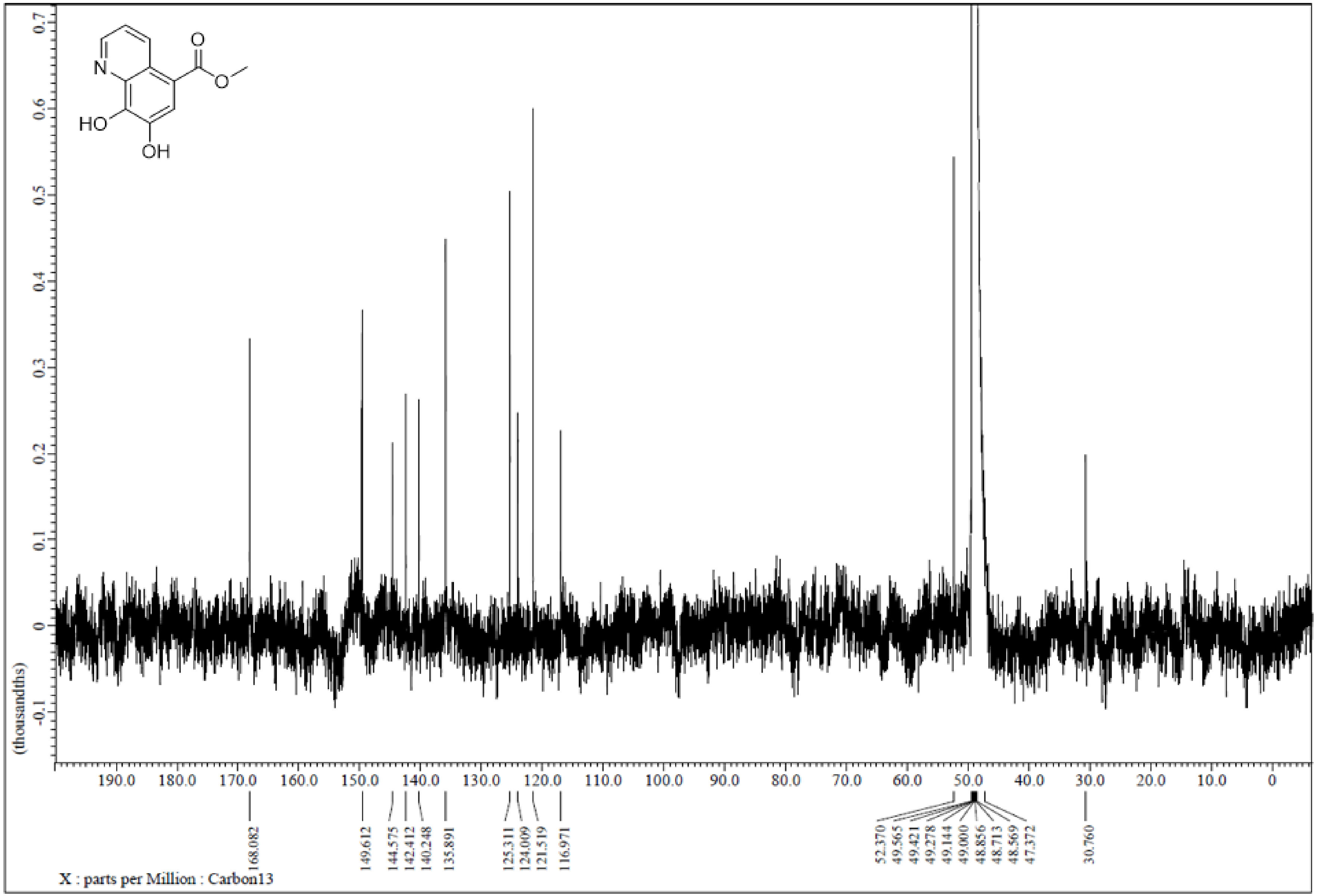
N
O
O
HO
OH
0.7
0.6
0.5
0.4
0.3
0.2
0.1
0
-0.1
(thousandths)
190.0
180.0
170.0
160.0
150.0
140.0
130.0
120.0
110.0
100.0
90.0
80.0
70.0
60.0
50.0
40.0
30.0
20.0
10.0
0
168.082
149.612
144.575
142.412
140.248
135.891
125.311
124.009
121.519
116.971
52.370
49.565
49.421
49.278
49.144
49.000
48.856
48.713
48.569
47.372
30.760
X : parts per Million : Carbon13

^{1}H-^{1}H COSY of 1

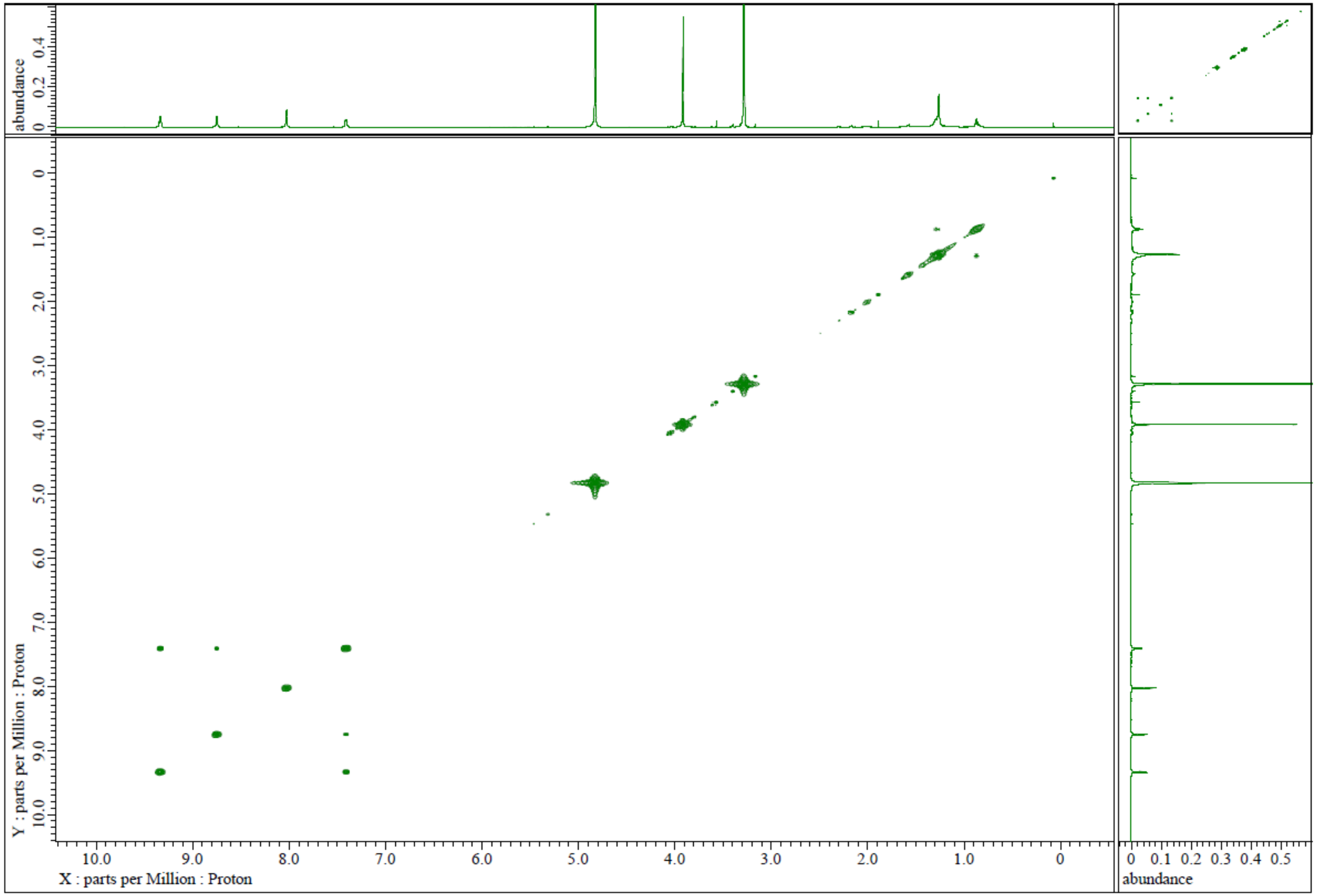

^{1}H-^{13}C HSQC of 1

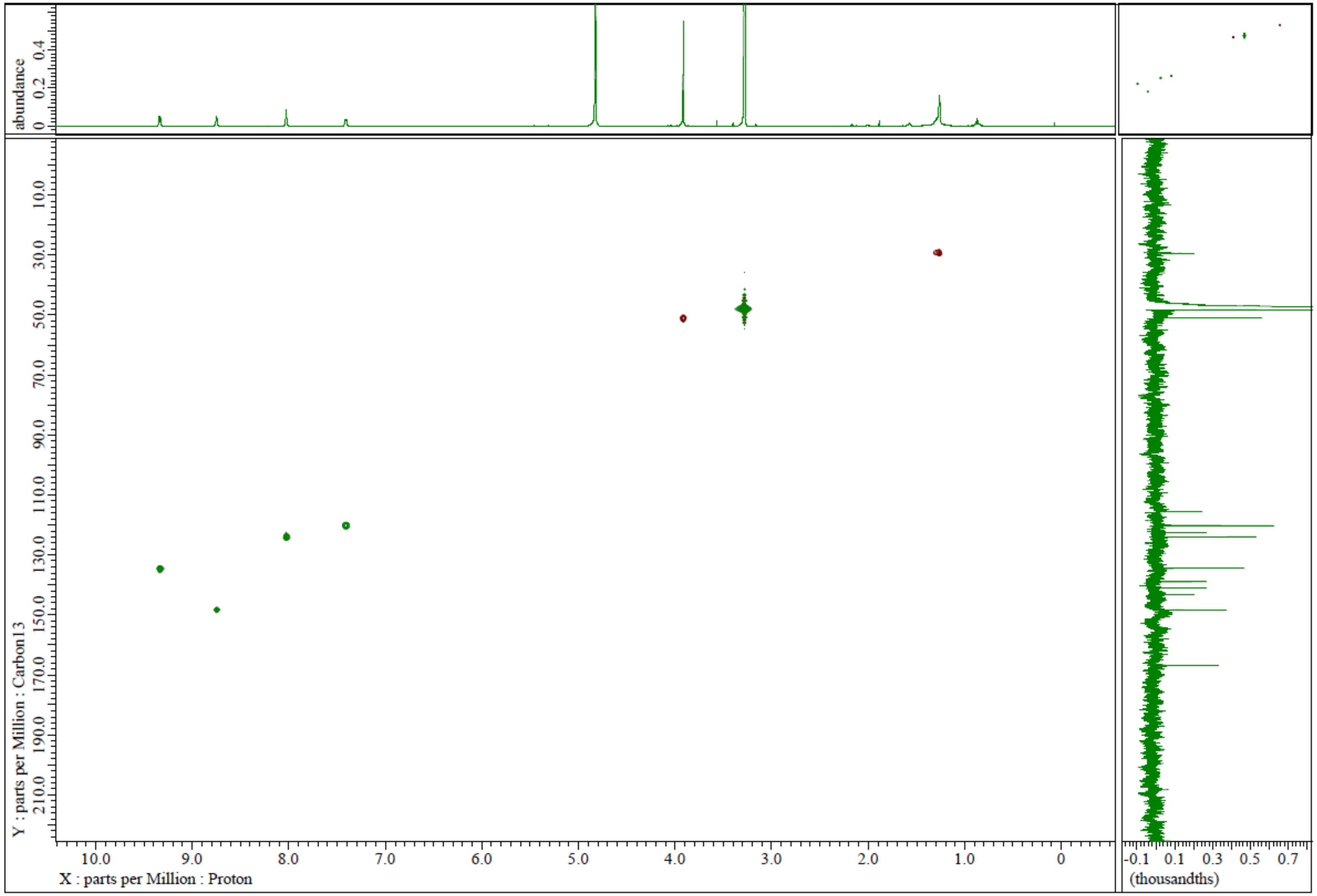

^1H-^{13}C HMBC of 1

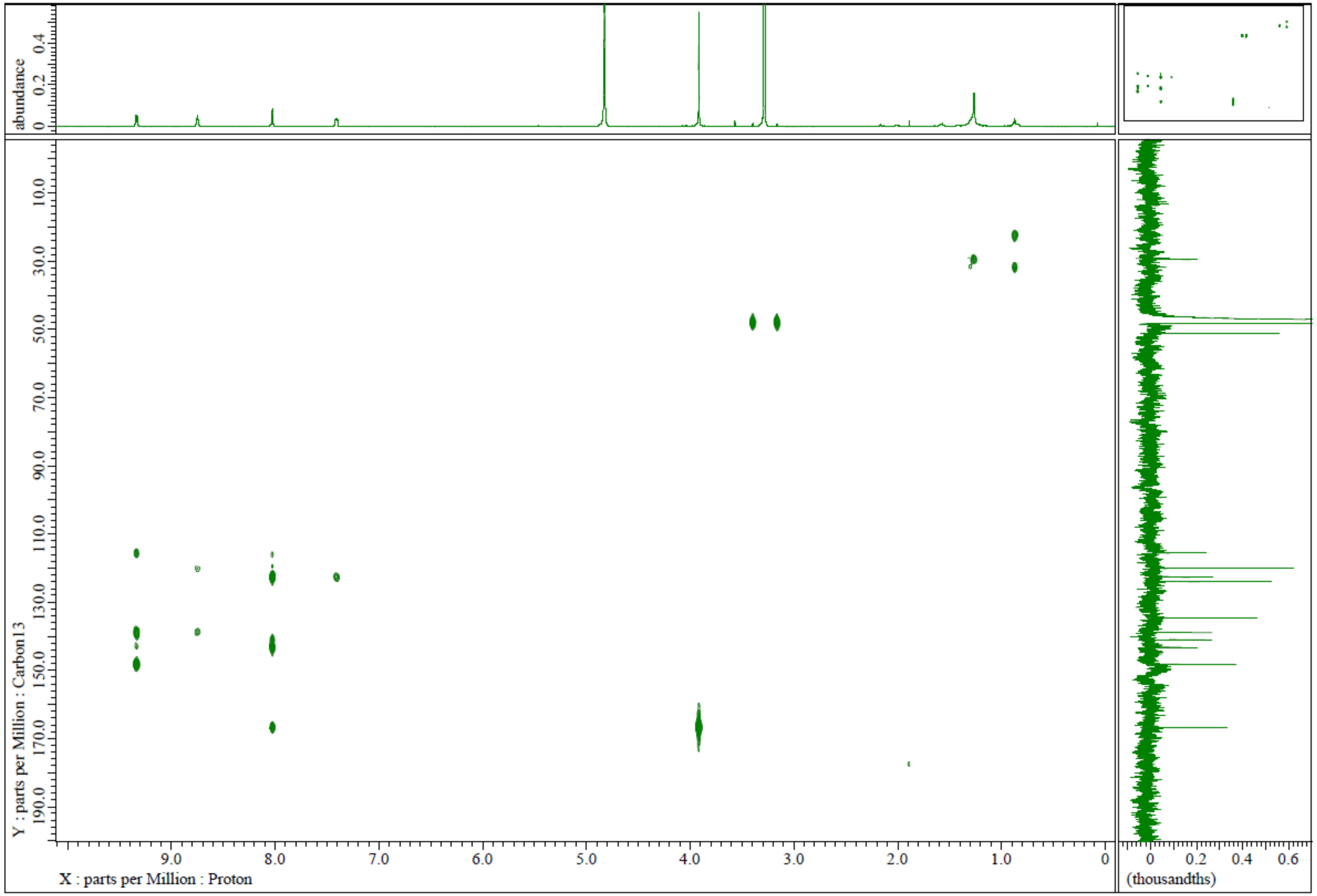

Supplement: Supplementary file 1 [file molecules-26-05964-s001.zip › molecules-1398743-supplementary.pdf]
